# Youth recidivism: youth self-report matters

**DOI:** 10.3389/fpsyg.2023.1208317

**Published:** 2024-01-04

**Authors:** Evan D. Holloway, Megan Irgens, Jeanne McPhee, Johanna B. Folk, Marina Tolou-Shams

**Affiliations:** Juvenile Justice Behavioral Health Lab, Division of Infant, Child, and Adolescent Psychiatry, Zuckerberg San Francisco General Hospital, University of California, San Francisco, San Francisco, CA, United States

**Keywords:** delinquency, multiple informants, youth, caregiver, recidivism, gender, measurement invariance, juvenile justice

## Abstract

**Introduction:**

“Recidivism” is used ubiquitously in juvenile justice research and typically describes repeat legal contact; however, researchers, policymakers, and clinicians operationalize it in various ways. Despite assuming each measure is a proxy for continued delinquent behavior leading to further legal contact, few have examined the association between youth delinquent behavior and self-reported and official records of legal contact. Furthermore, systemic bias against ethnoracial and gender minoritized youth often results in more harsh treatment by the legal system, which could influence recidivism measurement. Latent variable modeling of legal contact is understudied; thus, it is important to examine the feasibility of measuring this construct as a latent variable, including measurement invariance by gender.

**Methods:**

Among 401 youth ages 12–18 years at first ever court contact, we examined three metrics of legal contact over a 2-year follow-up period: youth-report of arrest, caregiver-report of their adolescent’s arrest, and official records of the number of new court charges. We examined between-group differences on each metric based on gender and ethnoracial identity. We then measured: (1) the association between youths’ self-reported delinquency and each metric, (2) gender-specific associations between self-reported delinquency and each metric, and (3) gender-based measurement invariance for a latent recidivism variable using confirmatory factor analysis.

**Results:**

Youth were consistent reporters of their own delinquent behavior and prospective legal contact measured by arrests. There were no between-group differences based on gender or ethnoracial identity for any legal contact measures. Delinquency and all legal contact variables were positively intercorrelated for the overall sample and the male subsample. For females, delinquency was not associated with caregiver-reported youth arrest or number of new charges. The latent legal contact variable had unique factor structures for male and female subsamples, suggesting no measurement invariance.

**Discussion:**

Youth-reported delinquency at first ever legal contact was most strongly associated with youth-reported arrest during a 2-year follow-up period, followed by caregiver-reported arrest, and the number of new charges. Unique latent variable factor structures for male and female subsamples suggests the inter-relation between legal contact variables is gender-specific. Stakeholders should consider prioritizing youth-reported delinquency since it is most strongly related to prospective youth-reported arrest.

## Introduction

Reducing delinquency and repeat contact with the legal system are primary goals of the juvenile legal system (Office of Juvenile Justice and Delinquency Prevention [OJJDP], 2023) given public health and safety implications of delinquent behavior. Given that participating in illegal acts is expected to increase the likelihood of repeat legal contact (i.e., recidivism), it is important to evaluate the relationship between delinquency and various measures of legal contact (e.g., re-arrest, new court charges) with findings informing policy, clinical work and research in this area.

Recidivism is a central outcome of interest in forensic psychology when studying youth involved in the legal system (henceforth referred to as “youth”). The National Institute of Justice (2022) defines recidivism as “a person’s relapse into criminal behavior, often after… sanctions or intervention for a previous crime” but is more commonly operationalized by researchers and clinicians as repeat contact with the legal system (e.g., re-arrest, new charges) after initial legal contact (Heilbrun et al., 2017). However, a lack of standardized operationalization of recidivism within the field continues to exist. For example, recidivism has been operationalized as new arrests, detention/placement/removal from the home, and charges filed in court or adjudications (Cottle et al., 2001). Differences across definitions such as these limits the ability to synthesize research to inform policy, prevention, and intervention efforts to keep young people out of the legal system.

Not only have researchers operationalized recidivism in a variety of ways (Cantor and Lynch, 2000; Thornberry and Krohn, 2000) they have also collected data from a variety of sources to measure the same construct, including official records, youth self-report, and caregiver report (Cottle et al., 2001). For decades, official records were considered the most accurate source of recidivism measurement. Yet, obtaining and interpreting official records for research purposes (e.g., Hawkins et al., 1977; Elliott, 1995) is challenging, and recent literature has highlighted limitations of relying solely on arrest records, including large proportions of missing data and bias associated with arrest rates (Piquero, 2020). For example, while race of the alleged perpetrator is often provided, police records inconsistently report ethnicity, leading to incomplete and biased numbers of offenses allegedly committed by Hispanic and Latinx people (Piquero et al., 2014). Furthermore, records of official arrests are likely biased by their base rates: Black youth are significantly more likely to be arrested than their white peers with similar delinquent behavior (Rovner, 2016). Consistent over-representation of and more punitive treatment toward ethnoracial minoritized youth is driven by structural racism within U.S. society, including “public policies, institutional practices, and cultural representations [that] operate to produce and maintain racial inequities” (Bonnie et al., 2013, p. 239), especially at the point of arrest. For example, Black communities are subjected to substantially higher rates of surveillance and policing in Black communities (Crutchfield et al., 2012; Fagan et al., 2016). Beyond concerns related to the accuracy of official records of legal contact data, they pose further challenges for researchers given idiosyncratic decision making and policies. For example, decisions to arrest, divert, detain, and adjudicate youth for delinquent behavior at various stages of court processing (see Heilbrun et al., 2017) varies by jurisdictions and likely over time within jurisdictions as well.

In recognition of the individual, system, and structural bias in arrest records, researchers began using self-report delinquency to replace or complement official records (see Krohn et al., 2010 for a review). However, the reliability and validity of self-report delinquency and arrest compared to other measures of legal contact for youth, such as peer-report of arrest and official records has long been debated (Huizinga and Elliott, 1986). Thus, the degree of agreement between self-report and official records of legal contact continues to be important and relevant to understand to inform empirically supported clinical services and policymaking, as well as to inform future research.

Researchers have found moderate agreement when comparing self-reported delinquency/arrest to official arrest records for adults (Maxfield et al., 2000; Payne and Piquero, 2016; Daylor et al., 2019) and for youth (Hindelang et al., 1981; Piquero et al., 2014; Jolliffe et al., 2016). However, data with 18–23-year-old males from the National Longitudinal Survey of Youth indicate that from 1979 to 1997, the relationship between self-report arrests and self-report delinquency became weaker when comparing the 18-year period between data collection points. Taken together, findings suggest that the association between delinquent behavior and contact with the legal system weakened significantly over time (Weaver et al., 2019). Therefore, it is important to prospectively examine the association between self-report delinquency and self-report arrests for youth who have already been in contact with the court and we are not aware of any that have examined consistency with caregiver report.

Few studies have examined (in)consistencies between youth self-report and official records based on race and ethnicity (Piquero et al., 2014) and gender (Farrington et al., 2009) despite evidence of differential treatment for girls (keep citations), and ethnoracial minoritized youth (MacDonald and Chesney-Lind, 2001; Gaarder et al., 2004; Zahn et al., 2010), and ethnoracial minoritized youth. For example, nationally representative data shows that Black and male youth who report not engaging in delinquent behavior are more likely to be arrested than white and female youth (Yelnur et al., 2021). For Black and Latino boys, being stopped by police leads to prospective increases in delinquent behavior, suggesting contact with police is iatrogenic (Del Toro et al., 2019). Black boys are also more likely to be re-arrested despite similar or lower levels of delinquent behavior compared to other boys of color (Del Toro et al., 2019). Furthermore, some evidence suggests that court-involved girls reported less delinquent behavior and were less likely to be charged with an aggressive offense than boys (Farrington et al., 2009).

In addition to self-report and official court records, caregiver report can also provide a valuable perspective regarding youth involvement with the legal system and behavioral phenomena that can lead to ongoing legal contact (Loeber et al., 1991). Research has shown that criminogenic risk factors reported by youth and their caregivers are differentially associated with recidivism. For example, caregiver report of their adolescent’s peer deviancy is associated with the number of new court charges youth receive on official records, while youth report is not (Holloway et al., 2022). In light of these findings, examining the consistency between different sources of recidivism data (youth- and caregiver-report, official records) or their association with youth-report delinquency is needed.

Given the limitations of solely relying on official records to measure youth recidivism noted above, the current study sought to examine the validity of self- and caregiver-report of arrest for youth following their first contact with the court by comparing youth self-report of arrest, their caregiver’s report of their arrest, and official records of new court charges over 2 years following their first ever court contact. The first aim was to describe the frequencies of youth-report delinquency, youth- and caregiver-reported arrests, and official records of new charges. We hypothesized the distributions would be similar across all measures of legal contact, with most of the sample reporting no legal contact, some reporting a few instances of legal contact, and very few youth reporting several instances of legal contact during the 2-year follow-up period. The second aim was to examine the predictive validity of youth-report delinquency (IV) on youth- and caregiver-report of youth arrests (DV1 and DV2, respectively), and official new court charges (DV3) data. We hypothesized that youth-report delinquency would be most strongly related to youth-report arrest. The third aim was to examine the feasibility of creating a legal contact latent variable. There was no hypothesis for this aim as it was exploratory. We created a latent legal contact variable (DV4) based on the arrest and court charge variables to compare the predictive validity of self-reported delinquency on the four legal contact variables. We also examined gender-based measurement invariance of the latent variable to examine utility of this approach for gender minoritized youth at first court contact.

## Materials and methods

### Participants and procedure

Data were collected from youth, aged 12–18, and caregiver dyads (*N* = 401) enrolled in a longitudinal study (Epidemiological Project Involving Children in the Court (EPICC); R01DA034538; PI: Tolou-Shams) and followed for 24 months at 4-month intervals soon after their first-time contact with a family court in a northeastern U.S. state (for more information about the parent study, see Tolou-Shams et al., 2019). For this sample, many youth did not have formal legal contact (e.g., arrest) before court contact. At each 4-month follow-up timepoint, various measures of legal contact were collected. Youth reported their delinquent behavior and youth-caregiver dyads reported the number of times the youth was arrested over the past 4 months. Official recidivism data was collected across the 24 months and the number of new charges during this time were included. Data for the current analysis are from all seven timepoints.

### Measures

#### Demographic variables

Youth were asked to report their gender (with response options of male, female, and non-binary),^1^ age, race, and Latinx ethnicity at baseline.

#### Youth self-reported delinquency

At every timepoint, youth reported their engagement in 23 delinquent acts [e.g., Have you hit (or threatened to hit) a teacher or other adult at school?; Have you broken into a building or vehicle (or tried to break in) to steal something or just look around?] during the past 4 months using the National Youth Survey of Self-reported Delinquency (Thornberry and Krohn, 2000). The number of unique delinquent acts reported at baseline were summed to create a count variable.

#### Self- and caregiver-report of youth arrest

Youth and caregivers were asked to report how many times the youth had been arrested in the past 4 months at each time point.

#### Official juvenile court records

The number of new charges filed during each 4-month period over the 24-month study were extracted from official court records. Some new charges were linked to an arrest while others were not.

The number of arrests and new charges were summed separately as three variables to reflect the degree of legal contact across the 24-month follow-up period. Arrest and new charge variables were highly skewed and kurtotic with most youth having zero new arrests or charges at every timepoint (see Table 1).

**TABLE 1:** Descriptive statistics for legal contact variables.

Legal variables	%	*M*	*SD*	Median	Range	Skewness	Kurtosis	*SE*
Youth-report arrest	9.73	0.67	3.49	0	0–56	12.00	170.42	0.17
Caregiver-report arrest	16.5%	0.32	0.86	0	0–5	2.98	8.56	0.04
Number of new charges	35.4%	1.07	2.33	0	0–16	3.41	13.55	0.12

#### Analytic plan

Data cleaning, wrangling, and analyses were conducted in R (R Core Team, 2023) and saved in a Quarto document that was uploaded to the Open Science Framework [https://osf.io/r92fa/?view_only=5af9f6cc06844803b28caafc0d8eac4b].

Descriptive statistics of dependent variables were calculated to compare the associations between youth-reported delinquency, youth, and caregiver report of the count of youth arrests, and the count of official records of new court charges across the 2-year follow-up period. Kendall correlations were calculated using the correlation package’s [correlation (method = “kendall”)] function (Makowski et al., 2020) to examine the associations between the IV and legal DVs for the overall sample and then separately by gender.

Between-group differences on legal contact DVs were assessed using the ggbetweenstats() function from the ggstatsplot package (Patil, 2021). A series of three generalized linear models using a negative binomial distribution were calculated with youth delinquency as the IV and each of the three legal contact DVs (youth report of their arrest, caregiver report of their adolescent’s arrest, and the number of new court charges). Youth age, ethnoracial identity, and gender were included as covariates. Receiver Operating Characteristic analyses reporting the Area Under the Curve (AUC) were calculated using the performance (Lüdecke et al., 2021). Based on prior findings from the EPICC dataset and other studies of legal contact and delinquent behavior for youth, all measures were expected to be right-skewed and not normally distributed.

To assess aim 1, the distribution of each variable was examined using density plots and descriptive statistics assessing skewness and kurtosis. Kendall’s non-parametric correlation with Holm’s adjustment for multiple comparisons was used to examine the strength and direction of association between self-reported delinquency at baseline (IV) and cumulative youth and caregiver report of arrests (DV1, DV2); and official records of number of new court charges (DV3) at the final 24-month follow-up period. As such, Kruskal-Wallis one-way ANOVA non-parametric tests were used to examine the differences between gender and ethnoracial groups on all three legal contact DVs.

For aim 2, a series of negative binomial regression models were used to test the association between self-reported delinquency (IV), and youth’s self-report of arrests (DV1), caregivers’ report of youth’s arrest (DV2), and official number of new charges (DV3). Covariates included ethnoracial identity, age, and gender. Area Under the Curve (AUC) Receiver Operating Characteristic (ROC) estimates measured the predictive validity of the IV (youth self-reported delinquency) on each DV.

The third aim was to examine the feasibility of creating a legal contact latent variable and examining between-gender measurement invariance. A legal contact latent variable was created via multi-group confirmatory factor analysis using the lavaan package (Rosseel, 2012). The CFA indicators were the youth self-report of arrest, caregiver-report of youth arrest, and number of official new court charges. Given significant non-normality (i.e., skewness, kurtosis) among the three indicator count variables, maximum likelihood with robust standard error method was used (see Table 1 for descriptive statistics for legal contact variables). To determine whether the legal contact latent construct was invariant (i.e., comparable across groups), we evaluated evidence of metric and scalar invariance between male and female subsamples. This was examined via a series of nested multiple-group models. Measurement invariance was tested using the lavTestLRT() function from the lavaan package (Rosseel, 2012) and measurementInvariance() function from the semTools package (Jorgensen et al., 2022).

Following Cheung and Rensvold’s (2002) guidance, a difference in the comparative fit index (CFI) greater than 0.01 between measurement invariance models suggests a worse fit and indicates the absence of measurement invariance. The chi-square test is increasingly sensitive as sample size increases and is inferior to CFI.

## Results

### Participant descriptives

See Table 2 for sample characteristics. Youth were between the ages of 12 and 18 years (*M* = 14.53, *SD* = 154). At baseline, about half of the sample had a first-time status offense (e.g., truancy; *n* = 194, 48.6%) and half a first-time delinquent offense (e.g., theft; *n* = 205, 51.4%). Youth identified their gender as male (*n* = 226, 56.8%), female (*n* = 170, 42.7%) or non-binary (*n* = 2, 0.01%). In this sample, youth identified as Latinx (*n* = 168, 42.4%), followed by White non-Latinx (*n* = 126, 31.8%), Black non-Latinx (*n* = 43, 10.9%), youth who selected “other” and non-Latinx and (includes all Asian racial identities; *n* = 31, 7.8%), multiracial non-Latinx (*n* = 28, 7.1%).

**TABLE 2:** Sample characteristics.

Demographic variables	*M* (*SD*)/*n* (%)
Age	14.53 (1.54)
**Sex**	
Male	226 (56.5)
Female	171 (42.7)
Non-binary	3 (0.01)
**Offense type**	
Status	194 (48.5)
Delinquent	206 (51.5)
**Race/ethnicity**	
White, non-Latinx	126 (31.7)
Black, non-Latinx	43 (10.8)
Other, non-Latinx	32 (8.1)
Multiracial, non-Latinx	28 (7.1)
Latinx	168 (42.3)

### Youth legal contact descriptives

**FIGURE 1:**
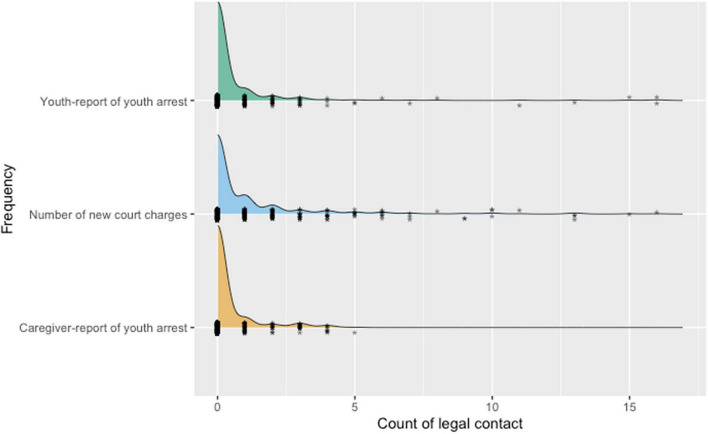
Density plot of legal contact variables. Each asterisk represents one data point.

Over one-third of caregivers and youth reported the youth had been arrested in the past 4 months at baseline (caregiver-report = 37.1%; youth-report = 37.4%). At the first follow-up 4 months after baseline, rates of arrest were lower (caregiver-report = 8.2%; youth-report = 13.2%); rates of arrest were lower than those at baseline and 4-month follow-up at each of the remaining 5 timepoints (caregiver-report range: 3.8% to 5.9%; youth-report range: 2.6–6.8%). Caregivers reported that youth had been arrested between 0 and 4 times at each follow-up timepoint while youth reported between 0 and 56 arrests. The youth self-report of arrest variable had two extreme outliers of 31 and 56 arrests. Since the next highest value under these outliers was 15, these values were recoded to 16 (maximum plus one). At each follow-up timepoint, between 7.3 and 13.5% of the sample received at least one new charge based on official records (range: 0–10 new charges). Density plots show similar distributions for all legal contact variables (see Figure 1).

### Relationship between legal contact variables

Results of Kendall’s correlation analysis showed that each of the legal contact indicators and self-report of delinquency were significantly correlated with each other (see Table 3). Given an interest in measurement invariance by gender, we examined correlations separately for males and females. All bivariate correlations between legal contact variables remained significant for both subsamples, however, self-reported delinquency was not correlated with caregiver-report of youth’s arrest or recidivism for females.

**TABLE 3:** Kendall correlations between independent and dependent variables.

Variable 1	Variable 2	*Tau*	95% CI low	95% CI high	*z*	*p*	*n*
**Overall sample**							
Youth-report arrest	Caregiver-report arrest	0.47	0.42	0.52	9.95	< 0.001	401
Youth-report arrest	Number of new charges	0.33	0.27	0.39	7.27	< 0.001	401
Youth-report arrest	Youth-report delinquency	0.24	0.18	0.30	5.46	< 0.001	387
Caregiver-report arrest	Number of new charges	0.46	0.41	0.51	10.14	< 0.001	401
Caregiver-report arrest	Youth-report delinquency	0.13	0.07	0.20	2.97	0.006	387
Number of new charges	Youth-report delinquency	0.11	0.04	0.17	2.50	0.012	387
**Female subsample**							
Youth-report arrest	Number of new charges	0.26	0.16	0.35	3.59	0.001	170
Youth-report arrest	Youth-report delinquency	0.20	0.10	0.30	2.97	0.009	166
Caregiver-report arrest	Number of new charges	0.44	0.35	0.52	6.12	< 0.001	170
Caregiver-report arrest	Youth-report delinquency	0.08	-0.02	0.18	1.18	0.475	166
Number of new charges	Youth-report delinquency	0.04	-0.07	0.14	0.53	0.594	166
**Male subsample**							
Youth-report arrest	Number of new charges	0.36	0.28	0.44	6.09	< 0.001	226
Youth-report arrest	Youth-report delinquency	0.26	0.18	0.34	4.55	< 0.001	218
Caregiver-report arrest	Number of new charges	0.47	0.40	0.54	7.90	< 0.001	226
Caregiver-report arrest	Youth-report delinquency	0.16	0.07	0.25	2.77	0.011	218
Number of new charges	Youth-report delinquency	0.15	0.06	0.23	2.61	0.011	218

### Legal contact variables by gender and ethnoracial identity

**FIGURE 2:**
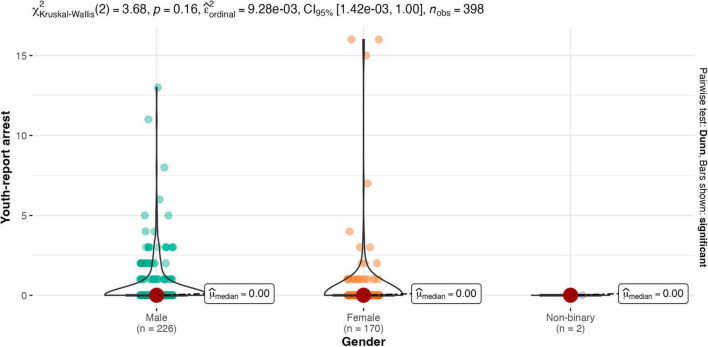
Gender by youth-report arrest.

**FIGURE 3:**
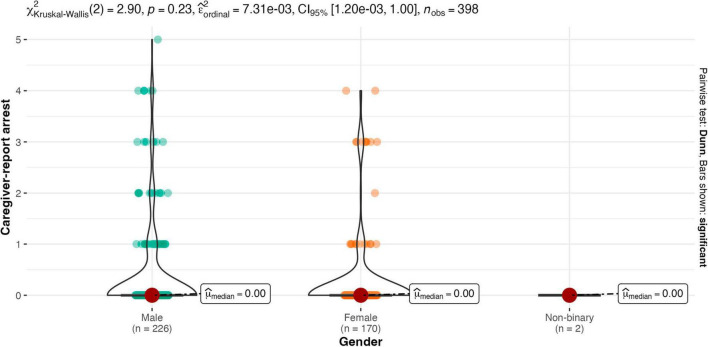
Gender by caregiver-report arrest.

**FIGURE 4:**
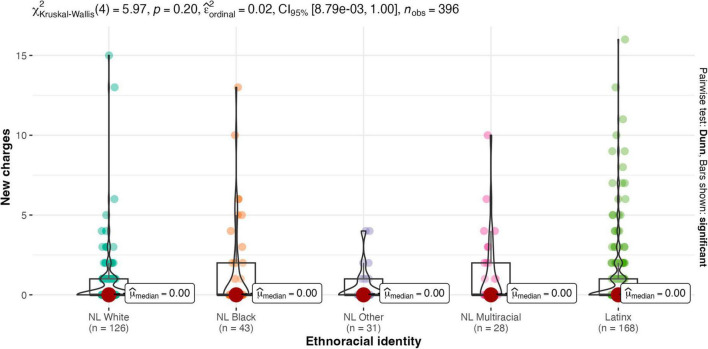
Gender by number of new charges.

**FIGURE 5:**
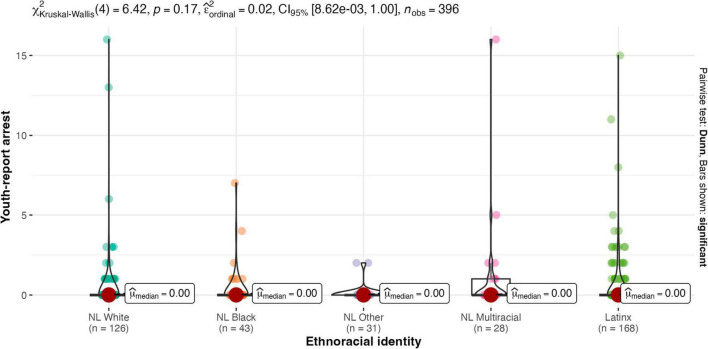
Ethnoracial identity by youth-report arrest. NL White, Non-Latinx White; NL Black, Non-Latinx Black; NL other, Non-Latinx other; NL Multiracial, Non-Latinx Multiracial; Latinx, Latinx only.

**FIGURE 6:**
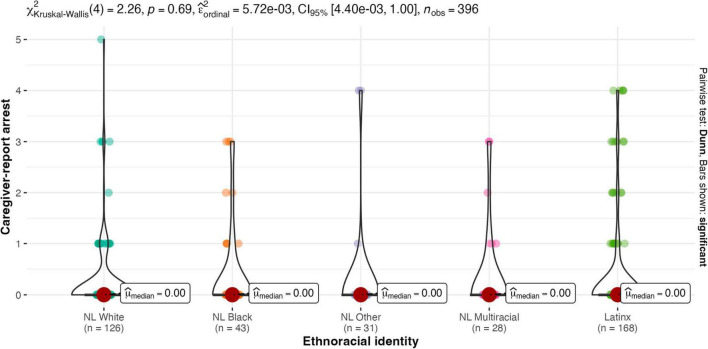
Ethnoracial identity by caregiver-report of youth’s arrest. NL White, Non-Latinx White; NL Black, Non-Latinx Black; NL Other, Non-Latinx Other; NL Multiracial, Non-Latinx Multiracial; Latinx, Latinx only.

**FIGURE 7:**
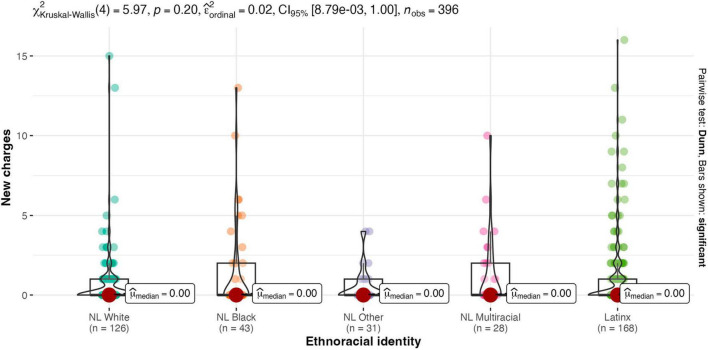
Ethnoracial identity by number of new charges. NL White, Non-Latinx White; NL Black, Non-Latinx Black; NL Other, Non-Latinx Other; NL Multiracial, Non-Latinx Multiracial; Latinx, Latinx only.

Results of the Kruskal-Wallis one-way ANOVA showed there were no between-group differences for males and females on any legal contact variable (see Figures 2–4). Similarly, there were no between-group differences in legal contact based on ethnoracial identity (see Figures 5–7).

The CFI for the configural invariance model (see Table 4 for standardized estimates) was 1.000 (df = 0) while the CFI for the metric invariance model was 0.977, suggesting no metric invariance (see Table 5 for measurement invariance results). This means the strength of the relationship between the three legal contact variables and the latent legal contact variable (factor loadings) is not the same (i.e., invariant) for males and females. The caregiver-report of arrest indicator had similar factor loadings for male (0.683) and females (0.686). However, the youth-report of arrest factor loading was stronger for males (0.531) compared to females (0.358) while the number of new charges loading was stronger for females (0.969) than males (0.691).

**TABLE 4:** Multigroup CFA results.

Parameter	Std. estimate	SE	Z	*p*	95% CI
**Female**					
**Factor loadings**					
Youth-report of arrest	0.531	0.158	3.371	0.001	0.222, 0.840
Caregiver-report of youth arrest	0.683	0.084	8.081	0.000	0.517, 0.848
Number of new charges	0.691	0.104	6.652	0.000	0.488, 0.895
**Variance**					
Youth-report of arrest	0.718	0.167	4.290	0.000	0.390, 1.046
Caregiver-report of youth arrest	0.534	0.115	4.629	0.000	0.308, 0.760
Number of new charges	0.522	0.144	3.633	0.000	0.240, 0.804
Legal latent variable	1.000	0.000	NA	NA	1.000, 1.000
**Intercept**					
Youth-report of arrest	0.359	0.035	10.353	0.000	0.291, 0.427
Caregiver-report of youth arrest	0.407	0.034	12.079	0.000	0.341, 0.473
Number of new charges	0.512	0.035	14.484	0.000	0.443, 0.581
Legal latent variable	0.000	0.000	NA	NA	0.000, 0.000
**Male**					
**Factor loadings**					
Youth-report of arrest	0.358	0.162	2.204	0.028	0.040, 0.675
Caregiver-report of youth arrest	0.686	0.100	6.889	0.000	0.491, 0.881
Number of new charges	0.969	0.108	8.980	0.000	0.758, 1.181
**Variance**					
Youth-report of arrest	0.872	0.116	7.518	0.000	0.645, 1.100
Caregiver-report of youth arrest	0.530	0.136	3.882	0.000	0.262, 0.797
Number of new charges	0.060	0.209	0.289	0.773	−0.350, 0.471
Legal latent variable	1.000	0.000	NA	NA	1.000, 1.000
**Intercept**					
Youth-report of arrest	0.165	0.026	6.256	0.000	0.113, 0.216
Caregiver-report of youth arrest	0.334	0.038	8.798	0.000	0.259, 0.408
Number of new charges	0.400	0.037	10.918	0.000	0.328, 0.472
Legal latent variable	0.000	0.000	NA	NA	0.000, 0.000

**TABLE 5:** Measurement invariance model comparisons.

Model	χ^2^ (Δχ^2^)	df (Δ df)	*p* (Δ p)	CFI (Δ CFI)
Configural invariance	0.000	0	–	1.000
Weak invariance (equal loadings)	7.07 (7.07)	(2)	0.029	0.977 (0.023)
Strong invariance (equal loadings and intercepts)	9.16 (2.08)	(2)	0.353	0.976 (0.000)

ΔCFI < 0.01 indicates that measurement invariance holds (Cheung and Rensvold, 2002).

### Predictive validity of youth-report delinquency on legal contact

Results of the ROC analyses showed that youth-reported delinquency at baseline was associated with each of the legal contact DVs (Ruscio, 2008). After accounting for the effects of age, gender identity, and ethnoracial identity as covariates, youth-report arrest was most strongly associated with youth-report delinquency (large effect size), followed by caregiver-report arrest (medium effect size), and number of new charges per official records (small-to-medium effect size; see Table 6).

**TABLE 6:** Area under the curve estimates for associations between youth self-reported delinquency and legal contact.

Legal contact DVs	AUC	Effect size
Youth-report arrest	0.742	Large
Caregiver-report arrest	0.636	Medium
New court charges	0.596	Small-medium

AUC = Receiver Operating Characteristic Area Under the Curve estimates; AUC conventions: Small = 0.560; Medium = 0.640; Large = 0.710 (Ruscio, 2008). Covariates included age, gender identity, and ethnoracial identity.

## Discussion

Measurement of continued contact with the legal system (i.e., recidivism) is of utmost importance for researchers and clinicians who study and work with youth involved in the legal system. While official court records have been considered more objective than self-report measures of legal contact (Hawkins et al., 1977), ample evidence of harmful bias against ethnoracial and gender minoritized youth warrants an examination of youth and caregiver-report of legal contact as alternative measures of recidivism.

This paper contributes to the literature by examining concurrent measurement of three measures of recidivism: youth’s report of arrest, their caregivers’ report of their arrest, and official new court charges during the 2-year follow-up period after initial court contact. We compared the distribution of each legal contact measure, between-group differences based on ethnoracial and gender identity, and each measure’s association with baseline youth self-reported delinquency; we also explored the feasibility of a latent legal variable for female and male youth by examining measurement invariance based on gender. Conclusions and implications are discussed below.

While most youth did not have subsequent legal contact across the 2 year follow-up period, according to all three measures, the distributions of youth report of their arrest, their caregivers’ report of their arrest, and the number of official records of new charges were similar. This finding is consistent with overwhelming evidence that youth in first-time contact with the court are at low risk for recidivism (Vincent et al., 2012; Hoge, 2016). However, measurement of each variable as binary (i.e., yes/no to any legal contact) or continuous (i.e., count of legal contact) tell different stories. For example, when considered as binary, official records resulted in the highest rates of recidivism, followed by caregiver-reported arrests of their adolescent, and youth-reported arrests. In contrast, when considered as continuous, youth self-reported arrests showed the widest range of legal contact, followed by the number of new court charges, and caregivers reporting the smallest range. It could be that some youth do not consider an experience with police as an arrest that their caregiver does. It is also possible, that those youth who experience relatively more arrests may have caregivers who are either unaware of all their youth’s arrests, do not consider them arrets, or under-reported them here, potentially due to recall bias or social desirability bias.

This finding supports the need to be specific and transparent in operationalizing the construct and measurement of recidivism, especially as this has larger implications for researchers synthesizing knowledge around specific types of legal contact (e.g., through use of meta-analyses). Furthermore, our findings suggest that official records may not be the most accurate measurement of problematic or illegal behavior and, therefore, should not be the only source used by legal and clinical practitioners who aim to provide targeted interventions to reduce and/or prevent recidivism and future delinquent behavior.

Our results showed differences in the number of youth and caregivers who reported youth being arrested. More caregivers reported the youth had ever been arrested than youth, suggesting that youth may either minimize the number of times they have been arrested, misunderstand the meaning of “arrest,” or struggle with retrospective recall (Huizinga and Elliott, 1986). Importantly, while just over half of the youth in the sample were referred to family court for delinquency petitions, only about one third of youth and caregivers reported an arrest in the 4 months prior to their baseline assessment. This may suggest that youth are being referred to court and/or being charged for crimes without being formally arrested and booked (e.g., due to school-based behaviors). This discrepancy may also indicate that youth may be scheduled for a court hearing more than 4 months after their arrest, which extends time and contact with the legal system and has implications for poorer overall youth outcomes, relative to youth in legal system for less time (Dir et al., 2021).

The range of arrests reported by youth and caregivers were also different, such that some youth reported far more arrests than their caregivers, even after removing outliers. This discrepancy may suggest that caregivers are not aware of the number of arrests that their children are experiencing. It may be that youth or legal actors (e.g., police, detention staff) exercise discretion when deciding when to inform caregivers their child has been arrested. Alternatively, this discrepancy may be explained by differences in how youth and their caregivers define arrest (e.g., stopped by police, handcuffed, confined in police car, or taken to police station). Qualitative research is needed to understand the phenomenology of legal contact by youth, their caregivers, and legal actors with whom they interact.

Our findings suggest that youth’s self-report of their delinquent behavior was most strongly associated with their self-report of arrest. Thus, if ongoing legal contact related to delinquent behavior is a primary outcome, then the current findings suggest that youth’ report of their own arrest is a better measure of ongoing legal contact than caregiver report of their arrest or the number of new charges because it is more strongly associated with their self-reported delinquent behavior.

Given well-documented worse legal outcomes among ethnoracial minoritized and female youth in the system, we examined whether there were between-group differences in each measure of legal contact. We found that the median count of legal contact was zero for all measures across ethnoracial and gender identities. In other words, the distributions of legal contact measures were comparable across ethnoracial and gender identities, beyond what is known in terms of over-representation of ethnoracial minoritized youth and worse outcomes for girls (MacDonald and Chesney-Lind, 2001; Piquero, 2008). However, our findings that youth-reported delinquency reported by females was not associated with all measures of ongoing legal contact, whereas it was for males, support the need for more research on sex-specific differences for measures of ongoing legal contact for youth.

When examining the relationship between each measure of legal contact and youth self-report of delinquency, we found that all variables were positively correlated with each other. When examining gender differences, these correlations held for males but not for females. Specifically, delinquency was not correlated with caregiver-report arrest or the number of new charges. This finding could reflect that we oversampled for status offenses in this sample and there is evidence that girls are over-represented among youth charged with status offenses, For example, national court processing data from 2019 showed females accounted for 44% of status offenses compared to 28% of delinquency cases (Puzzanchera et al., 2022). There is also evidence that girls are more likely to receive consequences for status offenses than boys (Mann, 1979), and Black girls receive more punitive legal consequences than white girls (Freiburger and Burke, 2011). The National Youth Survey of Self-reported Delinquency measure used in this study only included one item of 23 that measured a possible status offense (i.e., running away), suggesting that if girls were arrested for status offenses, this association would not have been captured by the current delinquency measure. Despite this limitation, the measure was chosen because it is commonly used in research, has evidence of external validity, and would permit comparisons with other research studies.

Low representation precluded examination of these relationships for non-binary youth. However, evidence suggests that sexual and gender minoritized youth have unique needs and may be subjected to worse outcomes (e.g., housing instability) due to their identity and systemic discrimination (see; Majd et al., 2009; Irvine, 2010; Irvine and Canfield, 2018). Nearly 30% of the current sample of youth reported sexual orientation and/or gender minoritized status (Hirschtritt et al., 2021).

We explored the feasibility of measuring legal contact through a latent variable approach. Given the unique patterns of contact with the legal system for male and female youth, we wanted to examine measurement invariance based on gender. We were able to do so because we intentionally oversampled for females, and therefore were able to examine gender- based measurement invariance to determine whether the factor structure of the latent variable was comparable for females and males in our sample. We did not find evidence for metric invariance (e.g., the factor scores were different) across male and female subsamples, suggesting the legal latent variable constructs were not comparable.

### Strengths, limitations, and future directions

The current study contributes to the existing literature by examining multiple measures of legal contact to inform researchers, clinicians, and policymakers about the external validity of youth-report of arrest, their caregiver’s report of their arrest, and official records of the number of new court charges filed. We also leveraged these data to explore the structure and gender-based measurement invariance for a latent legal contact variable using confirmatory factor analysis. Additionally, a major strength of this study is that we oversampled for subpopulations of youth who are under-represented in research, including females and those who became involved in the court due to a status offense.

Study data were collected from families at first court contact in a single state-wide jurisdiction in the Northeastern United States. Considering evidence of significant between-jurisdiction differences in juvenile legal system practice and decision-making (Knight et al., 2016; Holloway et al., 2017), these findings may not be generalizable to other jurisdictions and replication of these findings is encouraged. Another limitation is that we asked youth participants to identify their gender but provided responses consistent with biological sex (i.e., female, male, other) rather than gender (i.e., boy, girl). A related limitation is that the federal government reports biological sex rather than gender, which limits comparisons. Future research should confirm these findings with response options reflecting the gender identity spectrum more broadly and including a larger sample of gender expansive youth.

The minimum number of indicator variables per factor is three, which allows for a just-identified model with zero degrees of freedom (Wolf et al., 2013). Thus, measurement of a recidivism latent variable via confirmatory factor analysis requires at least three concurrent measures of legal contact. Since we oversampled for females, we were able to examine gender-based measurement invariance to determine whether the factor structure of the latent variable were comparable for females and males in our sample. We did not find evidence for metric invariance (e.g., the factor scores were different) across males and females, suggesting the legal latent variable constructs were not comparable, and no further analyses were conducted. While important to assess measurement invariance based on ethnoracial identity, our sample’s small sample size within ethnoracial identity groups did not allow for this analysis. Future research on recidivism as a latent construct should measure additional measures of legal contact so that the models have more degrees of freedom. Researchers should consider sampling strategies that allow for more balanced numbers of youth from different ethnoracial identities to allow for an examination of measurement invariance between groups.

### Implications

When considered as binary measures of recidivism, official court records may indicate higher rates of legal contact while caregiver- and youth-report of arrest may indicate less. Our findings suggest that researchers, clinicians, and policymakers should not consider each measure of recidivism or legal contact to be measuring the same construct and should consider the implications when selecting or evaluating measures of recidivism. For example, if measuring prior contact with police as a potentially traumatic event or as increasing iatrogenic risk (see Del Toro et al., 2019), youth-report of arrests may be an ideal measure of legal contact. In contrast, if continued court involvement is perceived as increasing risk on a risk/needs assessment when a juvenile probation officer is making treatment decisions, considering official records of the number of official new charges will be key; this may be especially true because youth and caregivers may be likely to under-report their arrests in this context. Additionally, practitioners aiming to prevent future illegal behavior should consider using a measure of self-reported delinquency, as official arrest records may not capture behaviors addressable by targeted intervention. However, if each measure of legal contact is assumed as a proxy for or related to a youth’s delinquent behavior after initial court contact, the youth’s self-report of their own arrest should be considered.

Self-reported delinquency was correlated with all measures of recidivism for males, but not females. As such, recidivism for females in this sample was not related to their self-reported delinquent behavior, this finding should be replicated in other samples. These findings suggest that youth are consistent reporters of their own delinquent behavior and recidivism measured by arrests. Given systemic bias in who encounters the legal system, clinicians, researchers, and policymakers should heavily consider youth’s self-report of delinquent behavior as the most accurate way to measure recidivism when considering ways to prevent repeat legal system contact (i.e., recidivism).

## Data availability statement

The raw data supporting the conclusions of this article will be made available by the authors, without undue reservation.

## Ethics statement

The studies involving humans were approved by the University of California, San Francisco. The studies were conducted in accordance with the local legislation and institutional requirements. Written informed consent for participation in this study was provided by the participants’ legal guardians/next of kin and informed assent was provided by youth participants.

## Author contributions

MT-S obtained funding for this project and led the design and original data collection. MI led the data request and management process. MI, JM, and EH wrote the initial draft of the Introduction. JM wrote the initial draft of the Method. EH performed statistical analysis and led drafting of the manuscript. JF consulted heavily on statistical analysis and copyediting. All authors contributed to conceptualization, manuscript revision, read, and approved the submitted version.
